# Nonmetastatic nasopharyngeal carcinoma: in search of safer therapies

**DOI:** 10.1016/j.lana.2026.101420

**Published:** 2026-02-13

**Authors:** The Lancet Regional Health – Americas

Nearly 133,000 new cases of nasopharyngeal cancer and 80,000 attributable deaths occur globally each year. Although nasopharyngeal cancer is rare, less than one case for every 100,000 people each year, the incidence is considerably higher in China, south Asia, the Middle East, the Arctic, and north Africa. According to GLOBOCAN 2022 data, the incidence rate of nasopharyngeal cancer in the Americas was 0.44 per 100,000 person-years, with a total of 4593 new cases. Cuba had the highest rates of overall incidence and death in the Americas (1.5 and 0.95 per 100,000 person-years, respectively), whereas the USA had the highest number of new cases and attributable deaths (2008 and 858, respectively). Main risk factors include Epstein–Barr virus infection, family history, age, a diet high in cooked salty meat, and tobacco and alcohol use. Advanced stages have very poor prognosis, with a 5-year relative survival rate of 45%. However, for nonmetastatic cancer, the survival rate is higher (81%).

The management of nonmetastatic nasopharyngeal carcinoma (NPC) has evolved in the last decade. Intensity-modulated radiotherapy (IRTM) is the current standard treatment, with concomitant chemotherapy for stages II, III, and IV (nonmetastatic cancer). Intensity-modulated proton therapy (IMPT) has emerged as an alternative to IMRT. IMPT is a therapy based on a phenomenon in physics called Bragg peak, which consists of the delivery of the highest dose of radiation (protons) into the tumour before the particles come to rest, minimising the damage to non-cancerous tissues. Given the proximity of numerous anatomical structures to the nasopharynx, reducing and better targeting the radiation dosage to the nasopharyngeal tumour could potentially minimise damage to normal tissues.

Although IMPT for nonmetastatic NPC appears to be associated with lower acute toxicity compared with IMRT, it is unclear whether IMPT is associated with lower long-term toxicity. To answer this question, Cao and colleagues did a case–control study to evaluate 159 patients with NPC treated with IMPT or IMRT at Memorial Sloan Kettering Cancer Center, with a median follow-up time of 55.4 months. Analyses included ten acute complications and 14 long-term complications, among which were dry mouth, dysgeusia (taste distortion), hearing impairment, and temporal lobe injury. The lower incidence of moderate acute toxicity (grades 3 or 4) with IMPT compared with IMRT was clinically meaningful. However, the incidence of moderate long-term toxicity (grade 3) was not significantly different between therapies. This study also found no significant differences in overall survival between therapies at 2 (IMRT 96.6% and IMPT 93.5%) or 5 years of follow-up (IMRT 87.2% and IMPT 85.6%).

A case-matched study in individuals receiving IMPT or IMRT for oropharyngeal cancer, another indication for intensity-modulated radiotherapy, also reported no differences between IMPT and IMRT in overall survival or progression-free survival after a median follow-up of 32 months. And a preliminary report of a phase 3 clinical trial (TORPEDO) in 205 individuals with oropharyngeal cancer from the UK also found no statistically significant differences in quality of life in the long-term after treatment with IMPT or IMRT. 2-year overall survival was similar. The TORPEDO trial and the two other observational studies found no evidence that IMPT provides any additional benefits over IMRT to reduce long-term toxicity or improve survival in individuals treated for NPC or oropharyngeal cancer. However, all three studies showed reduced acute toxicity with IMPT.

The study by Cao and colleagues is the longest and most comprehensive prospective study published to date comparing toxicity between IMRT and IMPT for NPC. While the findings suggest that IMPT may reduce acute toxicity compared with IMRT, it is disappointing that no additional benefits on long-term toxicity or survival were observed with IMPT. A possible explanation could be the low incidence of moderate to severe long-term toxicity with both therapies. Another aspect that should not be overlooked is the cost. Most NPC cases occur in regions where resources are limited. IMPT is more costly than IMRT and less widely available.

Good progress has been made with the development of IMPT for NPC; however, its efficacy and safety should be evaluated in a randomised clinical trial. The clinical implications of safer therapies for NPC are obvious. If clinical trials confirm the efficacy and safety of IMPT, cost-effectiveness studies should determine whether the clinically meaningful lower incidence of acute toxicity with IMPT compared with IMRT for NPC is justified. Strong evidence from clinical trials would accelerate improvements in the quality of life of individuals with NPC or further stimulate the development of safer alternative therapies with better efficacy in long-term overall survival.

